# Role of Human Corneal Stroma-Derived Mesenchymal-Like Stem Cells in Corneal Immunity and Wound Healing

**DOI:** 10.1038/srep26227

**Published:** 2016-05-19

**Authors:** Zoltán Veréb, Szilárd Póliska, Réka Albert, Ole Kristoffer Olstad, Anita Boratkó, Csilla Csortos, Morten C. Moe, Andrea Facskó, Goran Petrovski

**Affiliations:** 1 Stem Cells and Eye Research Laboratory, Department of Ophthalmology, Faculty of Medicine, University of Szeged, Szeged, Hungary; 2 Center for Clinical Genomics and Personalized Medicine, Department of Biochemistry and Molecular Biology, University of Debrecen, Debrecen, Hungary; 3 Department of Medical Biochemistry, Oslo University Hospital and University of Oslo, Oslo, Norway; 4 Department of Medical Chemistry, University of Debrecen, Debrecen, Hungary; 5 Centre of Eye Research, Department of Ophthalmology, Oslo University Hospital, University of Oslo, Oslo, Norway

## Abstract

Corneal tissue regeneration is of crucial importance for maintaining normal vision. We aimed to isolate and cultivate human corneal stroma-derived mesenchymal stem-like cells (CSMSCs) from the central part of cadaver corneas and study their phenotype, multipotency, role in immunity and wound healing. The isolated cells grew as monolayers *in vitro*, expressed mesenchymal- and stemness-related surface markers (CD73, CD90, CD105, CD140b), and were negative for hematopoietic markers as determined by flow cytometry. CSMSCs were able to differentiate *in vitro* into fat, bone and cartilage. Their gene expression profile was closer to bone marrow-derived MSCs (BMMSCs) than to limbal epithelial stem cells (LESC) as determined by high-throughput screening. The immunosuppressive properties of CSMSCs were confirmed by a mixed lymphocyte reaction (MLR), while they could inhibit proliferation of activated immune cells. Treatment of CSMSCs by pro-inflammatory cytokines and toll-like receptor ligands significantly increased the secreted interleukin-6 (IL-6), interleukin-8 (IL-8) and C-X-C motif chemokine 10 (CXCL-10) levels, as well as the cell surface adhesion molecules. CSMSCs were capable of closing a wound *in vitro* under different stimuli. These cells thus contribute to corneal tissue homeostasis and play an immunomodulatory and regenerative role with possible implications in future cell therapies for treating sight-threatening corneal diseases.

The cornea is the transparent front part of the eye responsible for two-thirds of its refractive power. It serves as a first barrier against external pathogens. Up to 90% of the corneal thickness is composed of corneal stroma, which contains different types of cells packed between regularly stacked and equally spaced collagen fibrils. Viral, fungal, bacterial infections and injuries caused by physical or chemical agents can all cause corneal scar formation, which eventually leads to vision loss or blindness^1^,^2^,^3^. The damage of the corneal epithelial cell layer and the deeper stromal layer invoke a healing process mediated by activation of progenitor cells that are found in the limbal region of the cornea- the limbal epithelial stem cells (LESCs)^3^,^4^,^5^,^6^. These cells can be found in six limbal crypts ordered in special niches capable of differentiating into transient amplifying cells (TACs) and differentiated corneal epithelial cells (CECs)^7^,^8^,^9^,^10^. The regeneration of the cornea and the role of CECs play is not fully understood. It is hypothesized that TACs migrate centripetally and superficially during differentiation or, alternatively, the LESCs migrate to the site of injury^9^. LESCs can express mesenchymal stem cell (MSC)-like markers on their surface such as CD73, CD90 and CD105 and show potential for clonal expansion, however, these cells are distinct from MSCs^11^. LESC deficiency can lead to abnormal epithelial regeneration and visual loss^1^,^12^, but such deficiency in mice could not stop the corneal epithelial regeneration in the central part of the cornea, suggesting another type of progenitor/stem-like cells plays a role in the wound healing process^13^,^14^. Corneal stroma stem cells have been isolated from the limbal stroma of mice and differentiated into keratocytes, but no evidence exists whether these cells are MSC- or bone marrow-derived MSC(BMMSC)-like^13^. In humans, both CD34^+^ and CD34^-^ as well as CD105^+^ cells have been isolated from the corneal stroma, however, no data demonstrates the stemness and multipotency of these cells, nor has their specific immunosuppressive effect been shown^15^,^16^. Furthermore, nothing is known about the participation of corneal stroma stem cells in corneal tissue remodelling and immunomodulatory processes related to trauma or infections^17^.

In this study, we isolated and characterized human central corneal stroma stem cells and compared their genotype to LESCs and BMMSCs, as well as their surface marker phenotype to BMMSCs. In addition, their differentiation potential and the immunological and wound healing properties were tested *ex vivo* to possibly harvest such cells for future cell, immunosuppressive and wound healing therapy in humans^6^.

## Materials and Methods

### Cell cultures

Collection of corneal and limbal tissue and bone marrow samples complied with the guidelines of the Helsinki Declaration and was approved by the Regional Ethical Committee (DEOEC RKEB/IKEB 3094/2010 and 14387/2013/EKU-182/2013), which follows the EU Member States’ Directive 2004/23/EC on presumed consent practice for tissue collection^18^. Corneal buttons were removed from cadavers (Age: 72.3 ± 11.4 years, Sex: 13F/11M) within 24 hrs from death, then transferred into Dulbecco’s Modified Eagle Medium-(DMEM) (PAA Laboratories GmbH, Pasching, Austria) containing wells. Thorough rinsing with Betadine (Povidone-iodine solution, Purdue Pharma L.P. Stamford, Connecticut, USA) and PBS took place, after which the epithelium and the Bowman’s membrane were scraped off using a surgical knife; consequently, the corneal endothelium and the Descemet’s membrane were scraped off with the same method. Just the central part (approximately 6–7 mm diameter cube) of the cornea was used and cut into small square pieces. Grafts were plated to 24-well cell culture plates and cultivated in 1 mL DMEM-LG medium (DMEM Low glucose-containing medium, PAA Laboratories) supplemented with 10% fetal calf serum (FCS) (Gibco; Gibco, London, UK), and 1% Antibiotic-antimycotic solution (PAA Laboratories). Medium was changed every alternate day. Limbal tissue processing and isolation of LESCs has been described by our group previously^19^. For the isolation of BMMSCs, approximately 10 mL of bone marrow aspirate was obtained from the donors and then diluted by saline in a 1 to 3 ratio. The mononuclear cells were recovered by Ficoll Histopaque (Amersham Biosciences, Uppsala, Sweden) density gradient centrifugation. The number of live cells was determined by Trypan blue exclusion assay. Bone marrow nucleated cells (BMNC) were plated in 25 cm^2^ flasks at a density of 2 × 10^5^ living cells/cm^2^ and cultured in DMEM-LG medium, supplemented with 10% FCS and 1% Antibiotic-Antimycotic Solution (all obtained from PAA Laboratories). At passage 5, every cell culture was tested for antigen expression by flow cytometry, *in vitro* differentiation assays and absence of Mycoplasma (Lonza, Basel, Switzerland).

### Flow cytometry and immunochemistry

To analyze the expression of selected surface markers, three-color flow cytometry was used. The cells were stained on ice for 30 min with fluorochrome-conjugated antibodies, then measured on a FACS Calibur flow cytometer (BD Biosciences Immunocytometry Systems, Franklin Lakes, NJ). The data were analyzed using Flowing Software (Cell Imaging Core, Turku Centre for Biotechnology, Finland) and the results were expressed as means of positive cells (%) ± SD. For immunohistochemistry studies, cell cultures were fixed in 4% PFA. Cytoskeletal actin filaments were labelled by phalloidin-TRITC (Sigma-Aldrich, Budapest, Hungary) and the nuclei by Hoechst 33342 (Invitrogen, Oregon, USA). Samples were examined under an Olympus IX81 inverted microscope with MT20 station (Olympus, Münster, Germany) and Orca2 (Hamamatsu Photonics K.K., Japan) camera. Surface carbohydrate molecules were labelled with lectins (Vector Labs, Burlingame, CA) diluted in Hepes buffer (Sigma-Aldrich)and examined by Olympus FluoView 1000 confocal LSM (Olympus).

### Differentiation

To undertake trilineage differentiation, the isolated cells were applied to commercially available Gibco’s StemPro® Adipogenesis, Osteogenesis and Chondrogenesis Differentiation Kits (Gibco). All differentiation patterns were evaluated according to the manufacturer’s guidelines. Oil red O staining was used to detect the lipid-laden particles in the differentiated adipocytes. The mineral deposits during osteogenesis could be demonstrated by Alisarin red staining, while toluidine blue staining was used to label the chondrogenic mass formed by CSMSCs.

### Microarray data analysis

To compare the gene expression profiles of the different cells isolated, an Affymetrix Gene Chip Human Gene 1.0 ST Array (Affymetrix, Santa Clara, CA, USA) was used. 150 ng of total ribonucleic acid (RNA) was subjected to an Ambion WT Expression Kit (Thermo Fisher Scientific, Waltham, MA, USA) and a GeneChip WT Terminal Labeling Kit (Affymetrix) according to the manufacturers’ protocol, then washed and stained on FS-450 fluidics station (Affymetrix). The signal intensities were detected by Hewlett Packard Gene Array Scanner 3000 7 G (Hewlett Packard, Palo Alto, CA, USA). The scanned images were processed using GeneChip Command Console Software (AGCC) (Affymetrix) and the CEL files were imported into GeneSpring GX 12.6 software (Agilent Technologies Inc, Santa Clara, CA, USA). Robust microarray analysis (RMA) was applied for normalization. Based on the literature, stem cells-related genes were selected and statistical analysis was performed (One-way ANOVA with Tukey *post hoc* test and Benjamini-Hochberg FDR; fold change cut off being set at 2) to calculate p values and fold change.

### Activation of CSMSCs and quantification of cytokines released by ELISA

CSMSCs were seeded onto 24 well plates in 5 × 10^4^ cell/mL density. After 24 h of culturing, the cells were treated with ultrapure 1 μg/mL lipopolysaccharide (LPS) (InvivoGen, San Diego, CA, USA), 25 μg/ml Polyinosinic-polycytidylic acid (Poly:IC) (InvivoGen), 100 ng/mL tumor necrosis factor alpha (TNFα), 10 ng/mL interferon gamma (IFNγ) and 10 ng/mL interleukin 1 beta (IL-1β) (all from Preprotech, Rocky Hill, NJ, USA) in a fresh medium for additional 12 and 24 h. After the incubation, supernatants were harvested and kept at −20 °C till measurement. The concentration of the secreted cytokines was measured by an enzyme-linked immunosorbent assay (ELISA). BD OptEIA ELISA assay kits for IL-1β, interleukin-6 (IL-6), interleukin-8 (IL-8), interleukin-10 (IL-10), interleukin-12 (IL-12), IFNγ, TNFα and IFNγ-inducible protein 10/C-X-C motif chemokine 10 (IP10/CXCL10) were used following the supplier’s instructions (BD Pharmingen, San Diego, CA, USA).

### Western blot analysis

For this purpose, cells were cultured into 25 cm^2^ cell culture flasks. After activation with the appropriate cytokines and toll-like receptor (TLR) ligands (described earlier), the whole cell lysates were prepared by scraping cells in 1000 μL of ice-cold radioimmunoprecipitation assay (RIPA) buffer containing protease and phosphatase inhibitors (all from Thermo Fisher Scientific)according to the manufacturer’s recommendations. The lysates were centrifuged at 4 °C at 12000 g for 10 min to clear the cellular debris. Total protein was quantified using the Bradford protein assay kit (Sigma-Aldrich). Equal amounts of protein were separated by 10% SDS-PAGE electrophoresis and transferred to a nitrocellulose membrane using a semi-dry blotting system (Biorad, Hercules, CA USA) and labelled with the following antibodies: anti–glyceraldehyde-3-phosphate dehydrogenase (GAPDH) (1:5000; Covalab, Villeurbanne, France), nuclear factor of kappa light polypeptide gene enhancer in B-cells inhibitor (IκB) (1:1000; Cell Signaling, Danvers, MAUSA), p65 and p50 (nuclear factor kappa-light-chain-enhancer of activated B cells/NFκB;1:1000; Cayman Chemicals, Ann Arbor, Michigan, USA); matching horseradish-peroxidase (HRP)-conjugated species corresponding secondary antibody (1:10000; Sigma-Aldrich) was also used. Enhanced chemiluminescence system (Immobilion substrate, Millipore, Merck KGaA, Darmstadt, Germany) was applied to visualize the immunoreaction, which was then developed by Kodak X-ray film system (Kodak, Rochester, NY, USA).

### Mixed lymphocyte reaction and mitogen-induced cell proliferation

Peripheral blood mononuclear cells (PBMCs) were isolated by a Ficoll gradient centrifugation (Amersham Biosciences). Mitogen-activated T lymphocyte proliferation was induced by concanavalin A (ConA) or phytohemagglutinin (PHA, all from Sigma-Aldrich) used at a final concentration of 10 μg/mL and 1 μg/mL, respectively, added to 1 × 10^6^ PBMCs. CSMSCs were added to 1 × 10^6^ PBMCs at 10^4^, 2 × 10^4^ and 10^5^ cell numbers and co-cultured for 3 days. On day three, proliferation was detected by a BrDU colorimetric assay directly in the cell culture plate according to the manufacturer’s instructions (Roche, Budapest, Hungary).

### ECIS based wound healing assay

For studying wound healing *in vitro*, a standardized commercially available wound healing assay was used implemented via automated ECIS Zθ (Theta)system (Electric cell-substrate impedance sensing system, purchased from Applied BioPhysics Inc, Troy, NY, USA). Cells were cultured at 2 × 10^4^ density in the chamber of the ECIS electrode arrays (8W10E obtained from Applied BioPhysics). After cell inoculation, the wells were incubated overnight to form monolayers; wounding was performed by electroporation using voltage pulses with 40 kHz frequency, 3.5 V amplitude and 30 s duration. This led to the death and detachment of cells present on the small active electrode, which resulted in a standardized wound size which normally healed from the cells surrounding the small active electrode not being subjected to an elevated voltage pulse. Continuous impedance measurements started 2 h before wounding occurred and continued until 24 h. The following experimental setups were performed: i) cells treated with TLR ligands and pro-inflammatory cytokines and impedance measured without making a wound; ii) cells treated with TLR ligands and pro-inflammatory cytokines after making a wound; iii) cells treated with TLR ligands and pro-inflammatory cytokines before making a wound; iv) non-treated controls. All experiments were performed at least three times and in triplicates on three independent donors.

### Statistical analysis

Statistica 7.0 software (StatSoft Inc., USA) was used for the statistical analyses. Normality of distribution of data was tested by Kolmogorov-Smirnov and Lilliefors test. Non-normally distributed parameters were transformed logarithmically to correct their skewed distributions. R software was used for hierarchical clustering. Each experiment was performed at least three times and each sample was tested in triplicates. Data are expressed as mean ± SD or SEM. Statistically significant difference was determined with two way ANOVA analysis when there were more than two groups, while analysis between two groups was performed with a paired student-t test. A value of p < 0.05 was considered significant.

## Results

### Cell morphology and surface carbohydrate characterization

**Figure 1:**
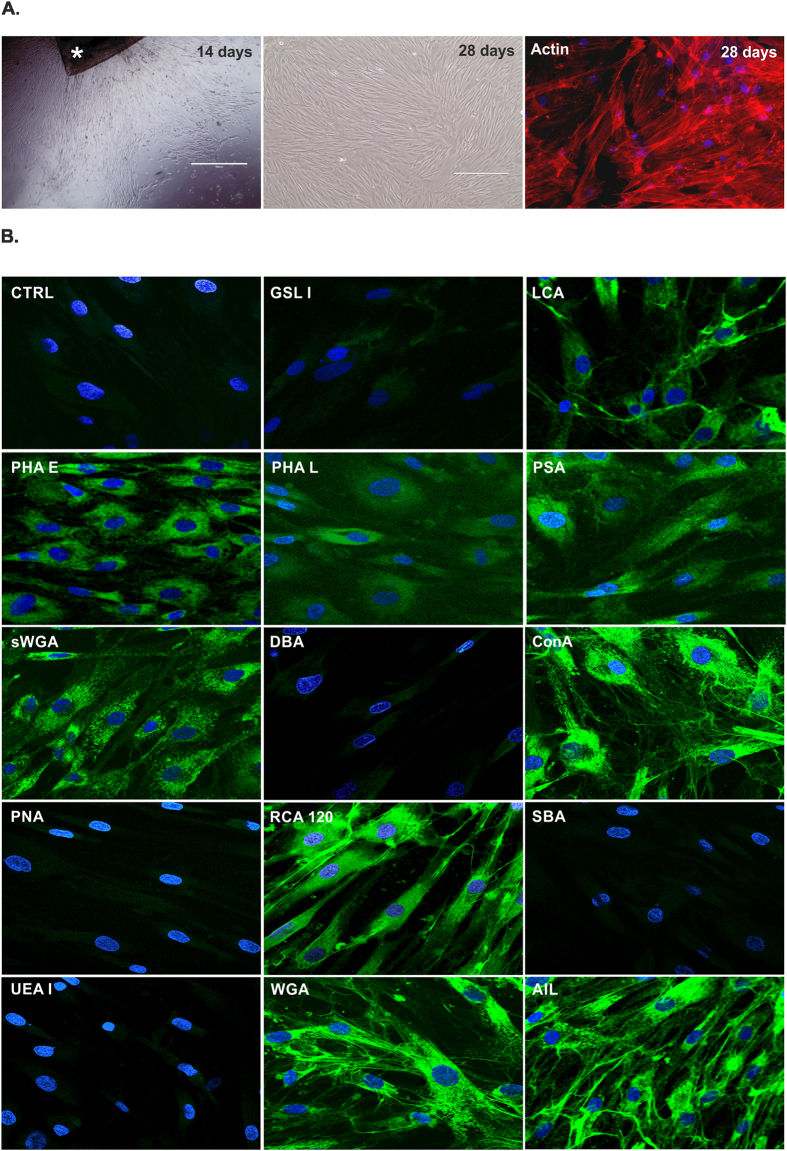
Morphology of the *in vitro* CSMSC cultures. Corneal stroma graft (*) isolated from the central part of the cornea was cultured in a cell culture plate (**A**), giving rise to elongated cells in the first 14 days of *in vitro* culture. Cells formed a monolayer by day 28, and showed fibroblastoid morphology according to their cytoskeletal structure. The expression of carbohydrate molecules on the surface of CSMSCs labelled by fluorescein-conjugated lectins is being shown (**B**). ***GSL I:***Griffonia (Bandeiraea) simplicifolialectin I (*Griffoniasimplicifolia*); ***LCA:***Lens culinaris agglutinin (*Lens culinaris*); ***PHA E:***Phaseolus vulgaris erythroagglutinin (*Phaseolus vulgaris)*; ***PHA L:***Phaseolus vulgaris leucoagglutinin (*Phaseolus vulgaris)*; ***PSA:***Pisumsativum agglutinin (*Pisumsativum*; s***WGA***: succinylatedWheat germ agglutinin *(Triticum vulgaris)*;***DBA:*** Horse gram lectin/Dolichosbiflorus agglutinin (*Dolichosbiflorus)*; ***ConA***: Concanavalin A *(Canavaliaensiformis)*;***PNA:*** Peanut agglutinin *(Arachishypogaea)*; ***RCA 120:***Ricinus communis agglutinin *(Ricinuscommunis)*;***SBA:*** Soy bean agglutinin (*Glycine max)*; ***UEA:***Ulexeuropaeus agglutinin *(Ulexeuropaeus)*; ***WGA***: Wheat germ agglutinin *(Triticum vulgaris); **AIL:***Jacalin*(Artocarpusintegrifolia)* (Magnification: 100X A1-3; 200XB1-14, Phalloidin-TRITC staining A-3, 400× Lectin staining B1-14).

Attached cells were detected after 10–14 days from human corneal stroma isolation in the culture plates as outgrowth from the graft, and formed a monolayer by 4 weeks (Fig. 1A). The outgrowing cells exhibited fibroblastoid morphology, assuming an elongated or spindle shape with a single nucleus. The established cell cultures could be maintained for more than 10 passages. At passage 5, cells were positive for RCA lectin which recognizes terminal galactose molecules (Fig. 1B). The two forms of Wheat germ agglutinins showed the succinylated and non- succinylated forms of dimer and trimer *N*-acetylgalactosamines on the isolated cells. High amount of mannose and D-glucose monomers and polymers could be detected by LCA, PSA, ConA and PHA-E (with *N*-acetylglucosamine) lectins, respectively. AIL labelled the β-galactose (1,3) *N*-acetylgalactosamine, which is the O-glycosidically linked oligosaccharide part of the T-antigen, while PNA negativity proved missing total T-antigen. The monomer form of *N*-acetylgalactosamine was not detected on the surface of the isolated and cultivated cells as DBA, SBA, GSL-I staining was similar to the unlabeled control. The well-known endothelial and epithelial marker UEA I (recognizing L-fucose) was also missing on these cells (Fig. 1B). RCA 120, ConA, WGA and AIL positivity could be detected more in the web-like structures between the cells, unlike LCA, sWGA and PHA-E which showed cellular localization. These findings could be verified by FACS measurements as well (Table 1).

### Gene expression analysis

**Figure 2:**
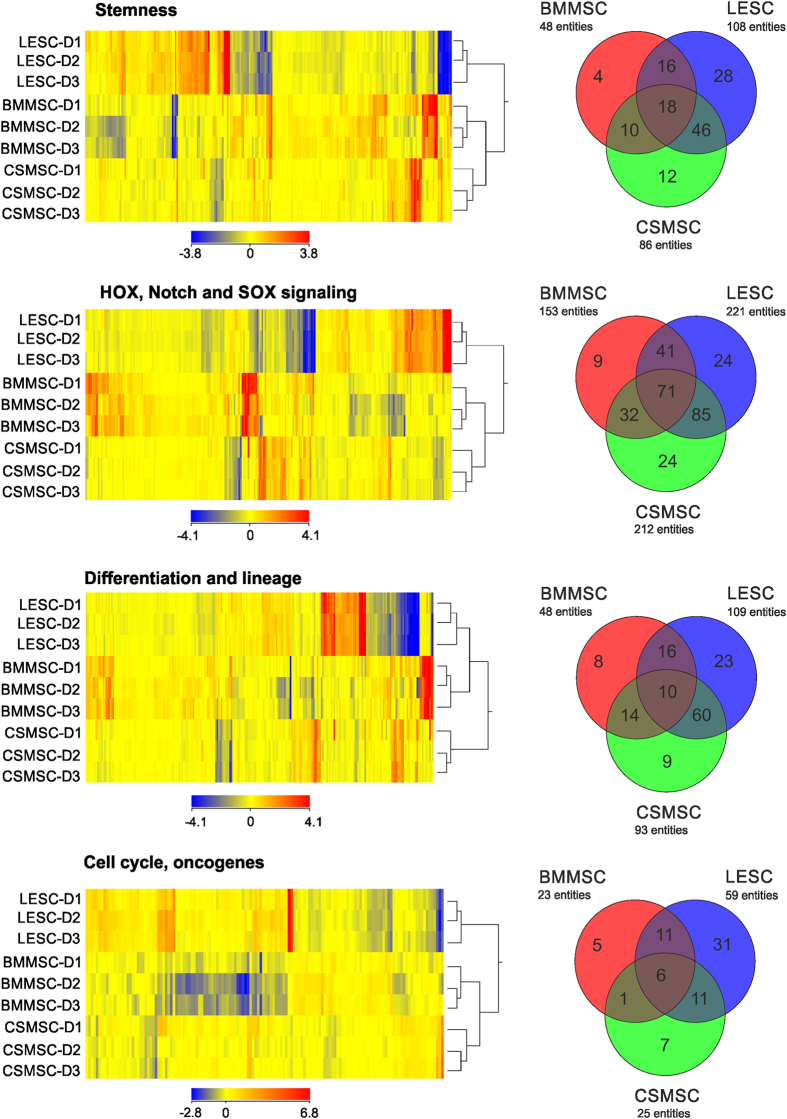
Heatmap of the differentially expressed genes in CSMSCs compared to LESCs and BMMSCs. Different expression levels of the transcripts and functional clustering of the genes expressed in *in vitro* cultured CSMSCs, LESCs and BMMSCs. Genes related to stemness, HOX, Notch and SOX signalling, differentiation and lineage, cell cycle and oncogenes were selected. The cluster analysis and dendrograms show the difference between the three cell types, and strengthen the finding that CSMSCs are more closely related to BMMSCs than to LESCs. Red and yellow colors indicate high and low expression, respectively.

Genes related to stemness (386), differentiation and lineage (468), cell cycle (220) and HOX, suppressor of cytokine signaling (SOCS), Notch signalling (372) were collected into functional groups and analyzed. The hierarchical clustering in CSMSCs clearly separated them from LESCs and BMMSCs, but formed a higher cluster with the BMMSCs (Fig. 2). In detail, an expression pattern of 12 genes related to stemness was found to be specific for CSMSCs, whereas *SLC48A1* (solute carrier family 48 member 1) and *DLL1* (delta-like1-Drosophila) expression was lower than in BMMSCs or LESCs. A significantly higher expression of *C12orf75* (chromosome 12 openreadingframe 75), *EDNRB* (endothelinreceptortypeB), *SMURF2* (SMAD-specific E3 ubiquitin proteinligase 2), *ACVR1* (activating A receptor type I), *TGFBR2* (transforming growth factor beta receptor II), *GATA2* (GATA binding protein 2), putative stem cell marker *ABCG2* (ATP-binding cassette sub-family G member 2), *LIFR* (leukemia inhibitory factor receptor alpha), *HSPA9* (heatshock 70 kDa protein 9/mortalin) and *CCND3* (cyclin D3) was determined as well. In the HOX, SOCS, Notch signalling superfamily, the CSMSC-specific pattern contained the following genes with low expression: *HES5* (hairy and enhancer of split 5-Drosophila), *WNT4* (wingless-type MMTV integration site family member 4), *WNT9A* (wingless-type MMTV integration site family member 9 A), *CCND2* (cyclin D2), *CREBBP* (CREB-binding protein), *KREMEN1* (kringle containing transmembrane protein 1), *CTNNB1* (catenin cadherin-associated protein beta 1), *FST* (follistatin), *EGR1* (early growth response 1), *MSX2* (msh homeobox 2), *PITX1* (paired-like homeodomain 1), *RUNX2* (runt-related transcription factor 2), *EGR3* (early growth response 3), and the following genes with high expression: *NFKB2* (nuclear factor of kappa light polypeptide gene enhancer in B-cells 2), *FOSL1* (FOS-like antigen 1), *LRP6* (low density lipoprotein receptor-related protein 6), *SEL1L* (sel-1 suppressor of lin-12-like-*C.elegans*), *TCF4* (transcription factor 4), *CALM2/CALM3/CALM1* (calmodulin 2/calmodulin 3/calmodulin 1 phosphorylase kinase delta), *IRS1* (insulin receptor substrate 1), *RBPJ* (recombination signal-binding protein for immunoglobulin kappa J region), *GJA1* (gap junction protein alpha1) and *MYC* (v-myc myelocytomatosis viral oncogene homologue), respectively.

The gene profile of CSMSCs in the differentiation and lineage custom group were: *MDK* (midkineneurite growth-promoting factor 2), *IGF1R* (insulin-like growth factor 1 receptor), *NTN1* (netrin 1), *RUNX1* (related transcription factor 1) and *DLL1* had lower expression compared to BMMSCs or LESCs; higher expression was detected in: *ACVR1* (activin A receptor type I), *TGFBR2* (transforming growth factor beta receptor II), *GATA2* (GATA binding protein 2) and *GDNF* (glial cell-derived neurotrophic factor).

Genes related to proliferation and cell cycle were distinct for CSMSCs and contained high levels of *NHP2* and *CCND3* (cyclin D3), low levels of *JUNB* (junB proto-oncogene), *BCL2L1* (BCL2-like1), *RUNX1*, *FHIT* (fragile histidine triad) and *CDKN2B* (cyclin-dependent kinase inhibitor 2B).

### Phenotype of CSMSCs

**Figure 3:**
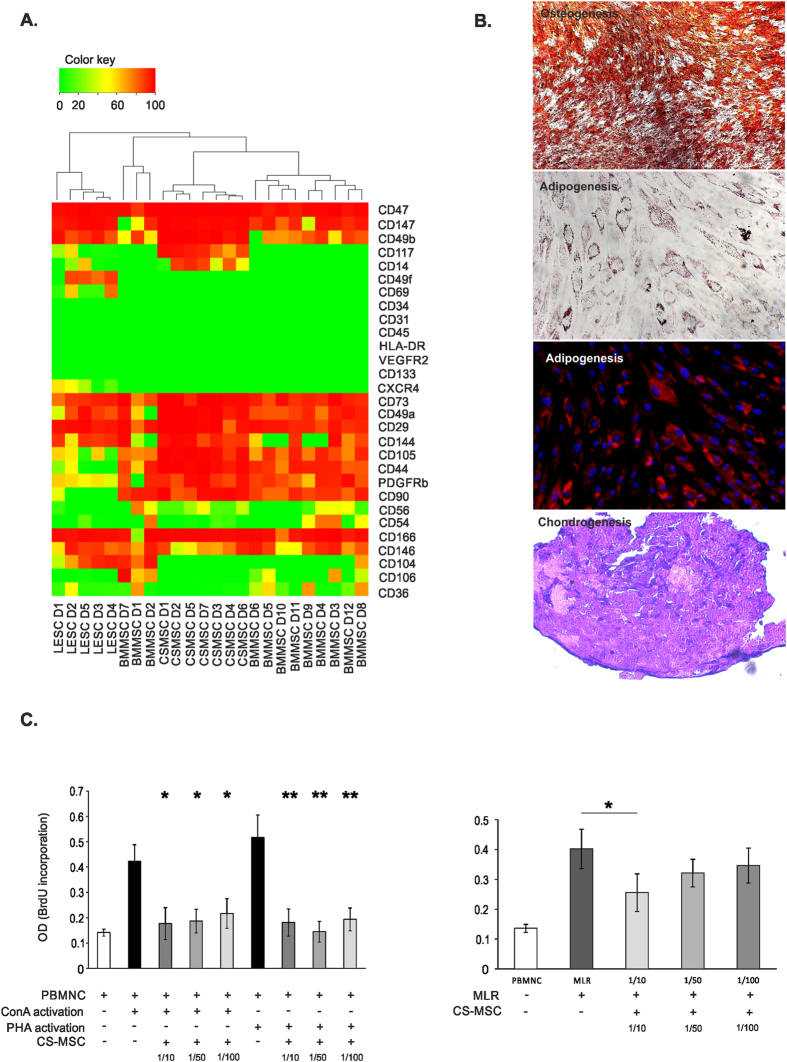
MSC-related phenotype, differentiation potential and immunosuppressive effects of *in vitro* cultured CSMSCs. Hierarchical clustering of cell surface molecules’ expression divided the stem cells of different tissue origin into two upper classes: CSMSCs were more closely related to BMMSC than to LESCs (**A**) (Color key represents percentage of positive cells in the *in vitro* cell cultures). CSMSCs were able to differentiate into the canonical mesodermal lines *in vitro.* Osteogenic differentiation shows the calcium deposits present as red-brown hue in the cell cultures visualized by Alizarin-red staining. Oil Red-O stained lipid droplet accumulations are shown in the adipocytes derived from CSMSCs *in vitro.* Metachromasia of the extracellular matrix stained by toluidine blue is shown in the cartilaginous section after chondrogenic differentiation (**B**). CSMSC could inhibit the proliferation of immune cells activated by PHA and ConA even in low cell numbers (left-hand panel) *in vitro*. Opposite to the mitogen-induced reaction, CSMSCs could block lymphocyte proliferation only at high doses in the mixed lymphocyte reaction (right-hand panel) (Data shown are mean ± SD, N = 3) (**C**).

Despite existence of the International Society for Cell Therapy (ISCT) defined most important markers of human MSCs, a clear MSC phenotype has not yet been well described. Our cells expressed the most important markers of MSCs such as CD73 (96.43 ± 3.88%, mean ± SD), CD90 (89.87 ± 8.80%), CD105 (76.99 ± 31.05%) and CD140b/PDGFRβ (76.63 ± 25.00%), but were negative for CD34, CD45, CD133, HLA-DR, which are markers of hematopoietic lineage or activated cells. Moreover, expression of other hematopoietic, fibroblast, endothelial-related markers, as well as integrins and cell adhesion molecules (CAMs) on the surface of CSMSCs were being determined (Table 2). No endothelial cells were detected within the CSMSC cultures, as VEGFR2, CD31 and CD104 were non-detectable. Furthermore, hematopoietic cell markers were absent (CD11, CD69) or the expression was low in the case of CD14 (1.39 ± 3.58%) and CD36 (5.80 ± 4.57%), respectively (the later results having a high interdonor variability being observed). Majority of the cells expressed CD49a/Integrin α1 (95.55 ± 7.15%), CD49b/Integrin α2 (98.05 ± 1.45%) and CD29/Integrin β1 (95.25 ± 6.95%). More than half of the cell cultures showed CD49d/Integrin α4 (61.75 ± 21.16%) positivity, few cells being positive for CD49f/Integrin α6 (3.03 ± 4.10%) and CD51/Integrin αV (16.62 ± 15.81%) besides their lack of CD104/Integrin β4 and CD18/Integrin β2 molecules. CAM molecules were highly expressed in the CSMSCs: CD44/H-CAM (96.07 ± 5.53%), CD166/ALCAM (93.44 ± 10.21%) and CD144/VE-Cadherin (94.56 ± 3.80%). A small subpopulation of the cells showed presence of CD325/N-Cadherin (27.72 ± 37.51%), CD146/MCAM (24.65 ± 24.97%), CD147/Neurothelin (69.49 ± 47.52%), CD54/ICAM-1 (24.79 ± 25.46%) and CD56/NCAM (17.21 ± 18.76%) positivity, however, huge interdonor variance was observed, respectively. Only a few cells were found to be CD106/VCAM-1 (2.11 ± 6.12%), CD112/Nectin (3.90 ± 8.85%) positive in some donors. CD117/c-kit (21.29 ± 34.25%) and CD338/ABCG2 (3.45 ± 5.03%) describe stem cell subsets and were found in some cases within the CSMSC cultures, but no cells could show measurable C-X-C chemokine receptor type 4 (CXCR4) expression. The high CD47 positivity (96.15 ± 7.02%) proved the viability and the immune competence of CSMSCs (Table 2). Comparative cluster analysis of the different surface markers showed that CSMSCs share similar phenotype to BMMSCs, and both cell types are different from the LESCs phenotype *in vitro* (Fig. 3A).

### Differentiation and immunomodulatory potential of CSMSCs

One of the hallmarks of human MSCs is their ability to differentiate into adipocytes, chondrocytes and osteocytes in culture. We assessed the *in vitro* differentiation potential of CSMSCs by culturing them into osteogenic, adipogenic, and chondrogenic media. During the chondrogenic differentiation, the cells formed a micromass pellet after 24 h. Following three weeks of differentiation, sections were made from the chondrogenic mass culture and showed metachromasia upon toluidine blue staining due to presence of proteoglycans (Fig. 3B). Fat globules could also be seen after three weeks of adipogenic induction stained with Oil Red ‘O’ (Fig. 3B). When induced with osteogenic induction medium for two to three weeks, the cultures showed mineral deposition indicating early stages of bone formation (Fig. 3B).

The immunosuppressive properties of MSCs have been extensively studied in the past years due to a major potential for clinical applications. In the present study, mitogenic MLR was used to test the immunosuppressive properties of CSMSCs. PBMCs from healthy donors were used as responder cells, and ConA or PHA as mitogenic activators. As shown in Fig. 3, the addition of CSMSCs to PBMCs and stimulation with ConA or PHA, suppressed the mitogenic response in a dose-dependent manner (Fig. 3C). In the MLR, no mitogenic activators were being used and only a high dose of CSMSCs was found to be immunosuppressive, whereas in low-doses, no anti-inflammatory function could be detected (Fig. 3C).

### Cytokine release from CSMSCs upon pro-inflammatory activation

**Figure 4:**
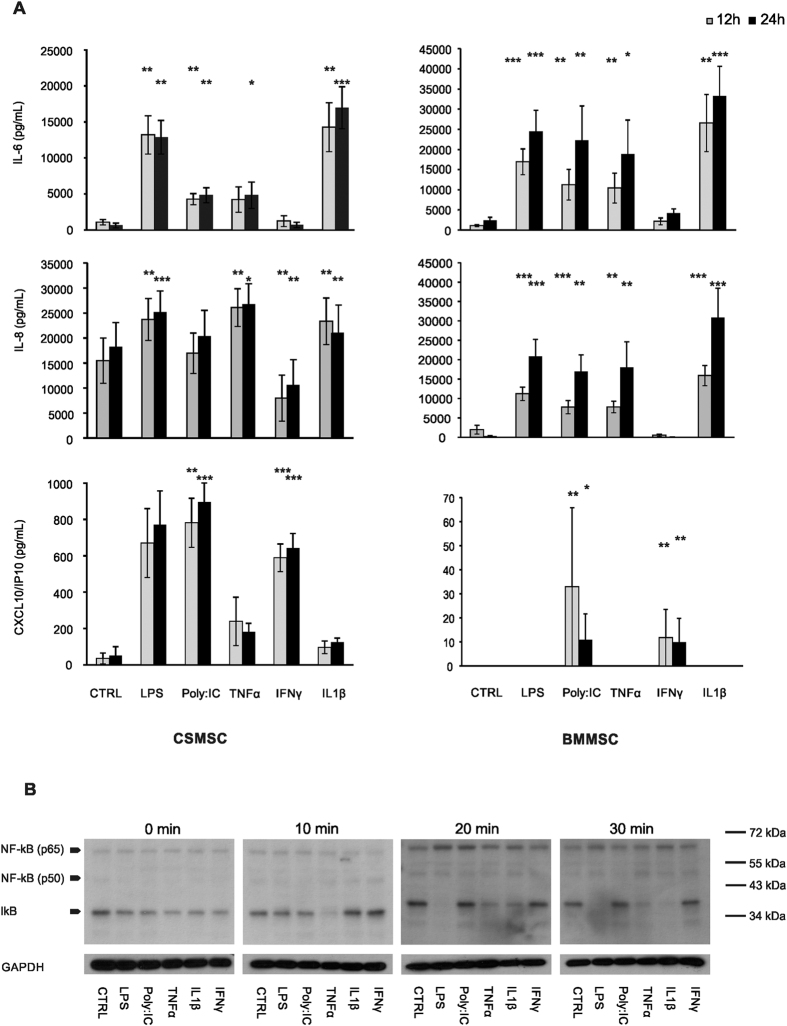
Cytokine secretion by activated CSMSCs. The bar graphs illustrate results of the quantitation of cellular responses of cytokine production by *in vitro* cultured CSMSCs and BMMSCs of both IL-6 and IL-8 and in response to activation by TRL ligands (LPS, Poly:IC), as well as pro-inflammatory cytokines (TNFα, IFNγ, IL-1β) observed after 12 h and 24 h intervals (**A**). Involvement of NFκB and IκB in the signalling pathways initiated by the TLR ligands and pro-inflammatory cytokines is being shown (**B**). (Data shown are mean ± SEM; *p < 0.05 **p < 0.01 ***p < 0.001 N = 6 for the BMMSCs and N = 9 for the CSMSCs, respectively).

The secreted cytokines’ response of *in vitro* cultured CSMSCs and BMMSCs was investigated upon TLR ligands (LPS and Poly:IC) or pro-inflammatory cytokines’ activation at different time points (Fig. 4A). IL-6 showed constant and continuous baseline secretion by both MSC types under control or untreated conditions, and increased significantly upon LPS treatment in CSMSCs (from 1098.98 ± 385.39 pg/mL to 13200.46 ± 2656.55 pg/mL at 12 h, and from 645.09 ± 327.64 pg/mL to 12883.07 ± 2335.30 pg/mL at 24 h time, respectively; data shown are mean ± SEM). Activation by Poly:IC, TNFα and IL-1β caused similar significant increase in the aforementioned baseline secretion of IL-6 by CSMSCs: 4295.81 ± 780.89 pg/mL (Poly:IC) and 14265.89 ± 3381.12 pg/mL (IL-1β) after 12 h, and 4843.41 ± 1049.07 pg/mL (Poly:IC), 4835.67 + 1836.90 pg/mL (TNFα) and 16974.47 + 2912.13 pg/mL (IL-1β) after 24 h, respectively. In case of IL-6 secretion, BMMSCs responded to all the treatments in the same manner as CSMSCs (Fig. 4A). Similar to the IL-6 response, however, the CXCL8/IL-8 and pro-inflammatory cytokines’ secretion was enhanced in CSMSCs upon TLR ligands treatment (Fig. 4A). Activation by LPS, TNFα and IL-1β caused even more robust and significant increase in the IL-8 release from the baseline secretion of 15472.92 ± 4532.47 pg/mL by the CSMSCs to 23710.37 ± 4195.08 pg/mL (LPS), 26084.64 ± 3765.65 pg/mL (TNFα) and 23349.24 ± 4656.87 pg/mL (IL-1β) after 12 h, and from the baseline secretion of 18253.36 + 4869.13 pg/mL to 25174.19 + 4255.29 pg/mL (LPS), 26756.89 ± 4104.02 pg/mL (TNFα) and 21066.17 ± 5529.87 pg/mL (IL-1β) after 24 h, respectively. Interestingly, IFNγ treatment caused a decreased release of IL-8 by the CSMSCs compared to the untreated control: from 7997.389 ± 4611.869 pg/mL after 12 h to 10631.81 ± 5042.48 pg/mL after 24 h, respectively, while such treatment did not show suppressed secretion by the BMMSCs. Poly:IC treatment did not alter the secretion of IL-8 by the activated CSMSCs, and the inverse effect was observed in BMMSCs (Fig. 4A).

**Figure 5:**
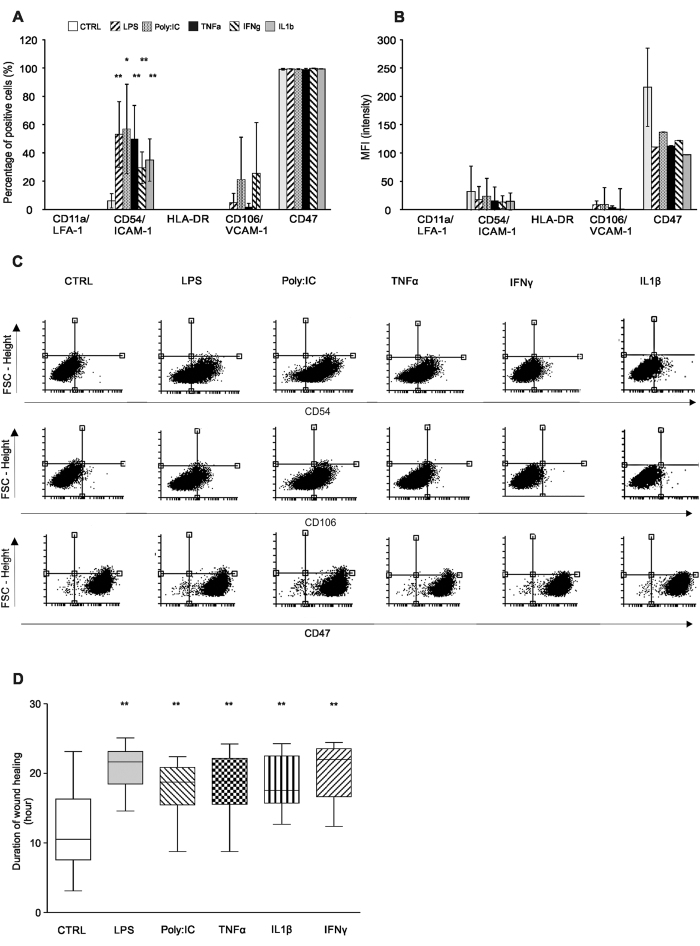
The effect of inflammatory stimuli on the CSMSC expression of cell adhesion molecules and wound healing ability. The percentage of positive cells for CD54/ICAM-1 increased upon pro-inflammatory stimuli within the *in vitro* CSMSC cell cultures (**A**). Median fluorescent intensity (MFI) changed just in the case of CD47 molecules (**B**). Dot plot of the selected surface markers is being shown (**C**). Impedance-based wound healing assay results investigating the wound healing properties of CSMSCs under normal and inflammatory conditions. Cells were treated after wound creation by pro-inflammatory cytokines and TLR ligands, which prolonged the time of wound closure as shown by the Whiskers boxes (**D**). (N = 6 donors in triplicates, *p < 0.05 **p < 0.01 ***p < 0.001).

Untreated CSMSCs secreted low levels of CXCL10 (35.26 ± 29.65 pg/mL at 12 h and 52.05 ± 48.28 pg/mL at 24 h, respectively), which could not be observed in the BMMSCs. This basic secretion level increased upon LPS, Poly:IC and IFNγ treatments (669.98 ± 189.45 pg/mL, 781.27 ± 134.70 pg/mL and 589.31 ± 75.90 pg/mL after 12 h, and 771.32 ± 184.87 pg/mL, 894.98 ± 105.82 pg/mL and 642.10 ± 81.36 pg/mL after 24 h, respectively) (Fig. 4A). No IL-1β, IL-10, IL-12, IL-17, TNFα and IFNγ production could be detected in the supernatants after the same treatments (therefore, not shown). The activation of CSMSCs was NFκB initiated: 10 mins after activation, only TNFα decreased the protein level of Iκb, which phenomenon could be detected in the 20 and 30 min samples. Treatments with LPS and IL-1β also resulted in diminished Iκb levels after 20 and 30 min of activation compared to untreated controls. The Poly:IC-dependent IL-6 and IL-8 secretion did not involve the IκB mediated pathway (Fig. 4B). Treatment of CSMSCs with inflammatory cytokines changed the level of cell adhesion molecules as well. The percentage of positive cells forCD54/ICAM-1 was significantly increased upon LPS (53.1 ± 23.22%, mean ± SD), Poly:IC (56.85 ± 31.63%), TNFα (49.8 ± 23.75%), IFNγ (29.43 ± 11.23%) and IL-1β (34.9 ± 14.93%) treatment (Fig. 5) when compared to untreated controls. In case of CD106, increased expression was observed, however, the high interdonor variability accounted for its non-significant increase by the Poly:IC and IFNγ treatments. No activation of HLA-DR or CD11a was observed and CD47 remained constant under all treatment modalities, although, its expression decreased upon pro-inflammatory stimuli (Fig. 5A–C).

### Wound healing properties of CSMSCs

The CSMSCs were able to close wounds showing robust regeneration activity under standard conditions. This phenomenon changed when cells were treated by pro-inflammatory cytokines or TLR ligands and following wound formation *in vitro*. The time of closing wounds was prolonged or, in some cases, inhibited the closure of the wound as was the case with LPS (20.89 ± 2.97 h, p = 0.000001, mean ± SD), Poly:IC (17.81 ± 4.07 h, p = 0.00282); TNFα (18.51 ± 3.95 h, p = 0.00108), IL-1β (20.19 ± 4.36 h, p = 0.00287) and IFNγ (18.27 ± 3.95 h, p = 0.00156) compared to non-treated cells with a wound (control, 12.37 ± 6.15 h) (Fig. 5D).

## Discussion

An isolation and cultivation protocol is hereby presented for harvesting and expanding MSC-like cells from the central part of the human corneal stroma with the use of FCS. The *in vitro* cultured cells fulfilled the ISCT criteria for MSCs^20^, as they were adherent to the cell culture plastic, could differentiate into three different lineages and expressed the desired MSC markers (CD73, CD90, CD105 and CD140b/PDGFRβ). The surface carbohydrate pattern of the corneal stroma stem-like cells, to date, has not been reported elsewhere. Previously, we published the carbohydrate fingerprint of *in vitro* cultured LESCs^19^, and in comparison to those cells, the *in vitro* cultured CSMSCs’ fingerprint differed significantly. The percentage of positive cells for the mannose and D-glucose binding ConA was higher in CSMSC cultures, with the concomitant decrease in UEA, PNA, DBA and SBA positivity. This different molecular pattern could reflect the different extracellular matrix niche of the two cell types. At gene expression level, the *in vitro* cultured CSMSCs expressed some putative LESC markers, however, their overall expression pattern was more similar to their bone marrow-derived counterpart, rather than the *in vitro* cultured LESCs. It also implicates that our CSMSCs are different and not related to the LESC niche, respectively. Based upon the literature and our data obtained, the finding that ABCG2 is not a true LESC-specific marker is hereby strengthened, as this gene is widely expressed by many other tissue-specific stem- and/or progenitor cells as well^21^. ABCG2 plays a key role in the fetal protection functioning as a xenobiotics transporter, as well as serves a protective function in the blood–brain barrier and the membranes of the hematopoietic and tissue resident stem cells^22^,^23^. This transporter is implicated as a Hoechst 33342 efflux pump, therefore, it is used to mark/or isolate the side population (SP-Hoechst negative) cells^21^,^24^. In corneal sections, ABCG2 labels SP cells in the limbus also (there being considered a putative marker)^11^. The detailed regulation of the expression of ABCG2, however, remains unclear as it is intensely controlled by the niche and the microenviroment of the cells^22^. In our dataset, it could implicate the size of the SP in the *in vitro* cell cultures. The amount of ABCG2^+^ cells in *in vitro* LESC cultures has been indeed controversial; there is a big variance between the used methods^25^,^26^,^27^; 2–4% of the LESCs cultured on mice feeder cells were found positive^28^, while the same amount could be reached with keratocyte medium cultivation as well^25^ (similar to our cultured CSMSCs), and in other model, the cell suspension and explant showed 50–85% positivity^27^. Interestingly, another ATP-binding cassette transporter (ABCB5) has been suggested to be an *in situ* specific LESC marker^29^,^30^, however, in our dataset, neither the *in vitro* cultured LESCs nor the CSMSCs expressed ABCB5. Interestingly, a weak expression could be detected only in one of the BMMSC donors (see Supplementary Fig. S1). As the expression of ABC transporters is mostly regulated by environmental factors, it is possible that the involved inducers are missing in the *in vitro* LESC/CSMSC/BMMSC cultures, respectively, as indicated by our data.

Further differences were observed in the surface molecular pattern between CSMSCs and LESCs. In comparison to previous publications from our department^19^,^31^, the expression of CD44, CD90, CD105, CD117, CD140 was higher-, and that of CD49f, CD104, CD146 and CXCR4 was less- in the MSC-like cells obtained from the central part of the cornea and compared to the LESCs. These differences indicate existence of different cell types within different microenvironments. In mice, MSC-like cells isolated from the central part of the corneal stroma exhibited similar phenotype except for a higher CD34 and CD45 positivity, with the canonical differentiation potential being shown there as well^32^. Due to the limited MSC markers studied in mice, a full comparison and correspondence to our results are not feasible^33^. These findings, however, strengthen the previous hypothesis, that stem cells exist not just in the limbal epithelial crypts, but independently, in the central part of the cornea as well^33^,^34^,^35^,^36^,^37^,^38^,^39^,^40^. The morphology of the *in vitro* cultured CSMSCs were fibroblastoid, not stellate or dendritic, which is characteristic of keratocytes^32^,^41^. Furthermore, keratocytes are known to be positive for CD34 and CD133markers, which were not expressed by the CSMSCs^42^,^43^. In fact, different culture conditions such as the type of the basal medium, the glucose concentration and the percentage or type of serum being used can cause changes in the phenotype and possibly an epithelial – mesenchymal transition^25^,^44^,^45^. However, change in the phenotype would not only affect the CD34 molecule, but CD105 and CD90 as well^44^, which has been connected to a possible epithelial-mesenchymal transition^25^,^46^. In our culturing system, the CSMSCs retained the CD90 and CD105 marker, while lacking CD34 expression over more than 10 passages. We would hereby like to highlight that the ISCT defined criteria for MSCs require absence of CD34 and presence of CD105, not accepting the CD34^+^ population as MSCs^20^,^47^,^48^,^49^,^50^.

The immunosuppressive properties of MSCs have acquired much interest in the last few years. As previous reports indicate, MSCs are able to stimulate or suppress immune responses *in vitro* and *in vivo* by multiple mechanisms^51^,^52^,^53^,^54^,^55^. Numerous studies have demonstrated that human MSCs avoid allorecognition, interfere with dendritic cells (DC) and T-cell functions, and generate a local immunosuppressive microenvironment^51^. MSCs are capable of inhibiting mitogen-stimulated lymphocyte proliferation *in vitro*^51^,^56^,^57^, and it is especially remarkable that our CSMSCs cultured *in vitro* possess similar immunosuppressive features, which phenomenon makes them potent candidates to treat ocular diseases with cell therapy. Such immunomodulatory effects could be observed and presumed *in vivo*^40^,^58^. While the immunosuppressive mechanisms of MSCs remain not fully clarified, the involvement of soluble factors, such as IL-6, IL-10, transforming growth factor β (TGFβ), IFNγ, Galectin-1, Leukemia inhibitory factor (LIF) and Prostaglandin E2 (PGE2) have strongly been suggested as mediators of anti-immunosuppressive response^59^. IL-6 is needed for PGE2 production and secretion, whereas PGE2 plays a key role in the inhibition of T-cell proliferation and DC maturation^55^,^59^,^63^,^64^,^65^,^66^. It is now accepted that modulation of DC maturation by MSCs requires IL-6 and a contact-dependent mechanism^63^,^67^. However, IL-6 can also act as a pro-angiogenic and migratory factor. Secreted by breast cancer cells in response to hypoxia, it increases the migratory effect of BMMSCs^68^. In clinical studies, elevated IL-6 concentrations could be measured within the aqueous humor from patients with endothelial rejection of corneal grafts, however, such findings could not be correlated with the rejection itself^69^,^70^,^71^. In rat corneal epithelial cells, the expression of HLA-DR and CD54/ICAM-1 increased upon TNFα and IFNγ treatments^72^, compared to our *in vitro* cultured CSMSCs in which changes in CD54 expression could only be detected, but none in HLA-DR. In human corneal epithelial cells, expression of MHC I-II proteins has been reported upon pro-inflammatory stimuli, which phenomenon could not be observed in our *in vitro* cultured CSMSCs. Keratocytes are sensitive to IL-1 and TNFα, which cytokines make them non-functional and apopototic - this phenomenon could not be observed in CSMSCs either^41^. Furthermore, keratocytes have not been described as immunosuppressive cells either, to the best of our knowledge^73^,^74^ - this function is only relevant to MSCs^50^,^75^,^76^. The outcome of the activation of MSCs by TLR ligands is controversial: activation by TLR3 and TLR4 ligands does not change their immune properties, but it inhibits their migratory effect as well as stimulates their adipogenic and osteogenic activity in case of TLR3 ligands; the opposite effect is observed in case of TLR4 ligands^77^. In another study, Poly:IC increased the cyclooxygenase 2 (COX-2) expression in umbilical cord-derived MSCs and enhanced the immunosuppressive effects on the macrophages, respectively^78^,^79^. In human corneal myofibroblasts, but not keratocytes, Poly:IC could provoke IL-6 and IL-8 secretion^80^. In contrast, the activation of TLR3 and TLR4 of BMMSCs leads to elevated inhibitory effect on T-cell proliferation, respectively^81^; furthermore, it enhances immunosuppression in another manner^18^.

CXCL10 has recently been found to be one of the most important cytokines expressed by human limbal epithelial progenitor cells at gene and protein level^82^,^83^,^84^,^85^. CXCL10 can act as a chemokine for MSCs^83^,^86^,^87^, and it has strong anti-angiogenic properties as well^88^,^89^,^90^. Our data suggest that this cytokine is probably specific for stem cells localized in the eye, while BMMSCs do not secrete it under normal conditions. CXCL10 secretion could be provoked by TLR-3, TLR-4 ligands and IFNγ in bone marrow- and eye-derived MSCs, which was found to be similar to other MSCs isolated from various tissues, respectively^81^,^91^,^92^. In corneal fibroblasts, LPS treatment elevated the CD54/ICAM-1 expression and IL-8 secretion, behaving similar to our CSMSCs^93^. The mechanism of corneal wound healing has not been clarified yet, however, it is clear that the process is controlled under various cytokines and growth factors^94^. In stromal cells from keratoconic corneas, the level of TNFα was found increased, which is supposed to lead to defects in wound healing^95^, inhibiting CEC migration and proliferation^96^. Similar results were obtained in a herpetic stromal keratitis animal model, where increased TNFα and IL-1β was correlated to the increased opacity of the tissue^97^. Alternatively, TNFα-treated MSCs were found to promote CEC survival, and inhibit apoptosis of these cells^98^. The lack of TNFα could elevate neovascularization and inflammation in an alkali-burn mouse cornea model^46^,^99^. Our *in vitro* cultured CSMSCs responded by elevated IL-6 secretion to TNFα exposure, which interleukin could be shown to promote corneal epithelial wound healing in a rabbit model^100^. IL-6 is strongly induced in alkali burns^94^,^101^, and supposed to be involved in the anti-inflammatory and anti-angiogenic action of MSCs in animal models of chemically burned corneas^102^,^103^ and during *in vitro* corneal wound healing modelling^104^. IL-1 can cause apoptosis of corneal myofibroblast, affecting physiological corneal wound healing^105^. Overall, the pro-inflammatory microenvironment can inhibit stromal cell function in the cornea, resulting in abnormal cell survival and wound healing, which eventually lead to rejection of allografts^106^,^107^.

Bacterial infections of the cornea can cause reduced vision, tearing, pain, and when left untreated, can cause blindness. The bacterial wall compound LPS is known to inhibit CEC migration and wound healing^108^, and to stimulate neovascularization and inflammation in the cornea^93^,^109^. The TLR4 and TLR9 affected signaling pathways are MyD88-dependentin animal models^110^, which complex system can also include the NFκB activation. Beside bacterial infection, viruses are able to provoke local immune and inflammatory responses in the cornea. Poly:IC increases the expression of vascular cell adhesion molecule (VCAM) and intercellular adhesion molecule -1 (ICAM-1) in human corneal fibroblasts^111^,^112^, while elevated secretion of IL-6, IL-8, IP-10 levels^111^,^113^ have been found to mediated by the NFκB pathway^111^,^112^,^113^, and similar to our results. This pathway was responsible for triggered IL-8 secretion in human corneas after adenoviral infection^114^. CECs also release IL-6 and IL-8 upon NFκB modulation, again similar to how our CSMSCs behaved^115^. In sepsis, Poly:IC can improve the immunosuppressive function of MSCs via the TLR3 pathway^79^.

It is well known, that MSCs are not immunosuppressive in common, but need to be primed to achieve the unique immunosuppressive behaviour^62^. The physical, chemical or biological damage of the tissue is usually accompanied by a “priming” local inflammation^116^. The outcome of MSC-mediated wound healing and immunomodulation depends on the direct and indirect interactions with immune cells^116^. Prolonged inflammation inhibits the tissue regeneration potential of MSCs and leads to insufficient immunoregulatory activity^116^,^117^.

In conclusion, the central part of the human corneal stroma contains MSC-like cells with differentiation potential and possible immunosuppressive properties, suggesting that they can play an important role not just in the regeneration during tissue injury, but also in controlling the immune status of the microenvironment^37^. These properties make the CSMSCs plausible candidates for tissue engineering and future cell therapies for treating sight-threatening corneal diseases^118^.

## Additional Information

**How to cite this article**: Veréb, Z. *et al.* Role of Human Corneal Stroma-Derived Mesenchymal-Like Stem Cells in Corneal Immunity and Wound Healing. *Sci. Rep.* **6**, 26227; doi: 10.1038/srep26227 (2016).

## Supplementary Material

Supplementary Figure S1

## Figures and Tables

**Table 1:** Expression of carbohydrate molecules on the surface of *in vitro* cultured CSMSCs.

Lectin	Positive Cells (%)	Sugar Specificity
ConA	80.38 ± 5.36	α-mannose, α- D-glucose
GSL I	0.00 ± 0.00	α- D-galactose, α linked N-acetylgalactosamine
AIL	90.86 ± 1.85	(Sialic Acid)β1 D-galactose -3 α1 N-acetylgalactosamine
WGA	92.19 ± 2.34	α- or β-linked N-acetylglucosamine
UEA	0.00 ± 0.00	α1 L-fucose-2 D-galactose
SBA	8.15 + 5.66	β1 N-acetylglucosamine – 4 β1 N-acetylglucosamine, 4 N-acetylglucosamine - N-acetylneuraminic acid (sialic acid)
RCA	84.35 ± 6.11	α- or β-linked N-acetylgalactosamine
PNA	6.31 ± 5.68	β1 D-galactose −3 α1 N-acetylgalactosamine
DBA	1.10 ± 0.72	N-acetylgalactosamine
succinylated WGA	80.93 ± 8.21	N-acetylglucosamine
PSA	81.94 ± 6.26	α-mannose, α- D-glucose
PHA-L	78.00 ± 7.23	β4 D-galactose-4 β6 *N*-acetylglucosamine (β2*N*-acetylglucosamine - α3 mannose) α3 mannose
PHA-E	86.50 ± 3.76	β4 D-galactose - β2*N*-acetylglucosamine - α6 mannose (β4 *N*-acetylgalactosamine) β4 *N*-acetylgalactosamine - α3 mannose) – β4 mannose
LCA	84.00 ± 5.51	α-mannose, α- D-glucose

Majority of the CSMSCs contained mannose, glucose, poly-saccharides with N-acetylglucosamine and N-acetylgalactosamine molecules, and lacked fucose, based upon the specific lectin screening. These carbohydrate molecules could determine the extracellular matrix and/or cell-cell binding and immunological properties of the cells (Data shown are mean ± SEM, N = 8; for abbreviations see Fig. 1).

**Table 2:** Surface marker pattern of *in vitro* cultured CSMSCs.

Marker	Positive Cells (%)
CD11a (LFA-1)	0.00 ± 0.00
CD14	1.39 ± 3.58
CD18 (Integrin β2)	0.00 ± 0.00
CD29 (Integrin β1)	95.25 ± 6.95
CD31 (PECAM)	0.00 ± 0.00
CD34	0.00 ± 0.00
CD36	5.80 ± 4.57
CD44 (H-CAM, Hermes)	96.07 ± 5.53
CD45	0.00 ± 0.00
CD47	96.15 ± 7.02
CD49a (Integrin α1)	95.55 ± 7.15
CD49b (Integrin α2)	98.05 ± 1.45
CD49d (Integrin α4)	61.75 ± 21.16
CD49f (Integrin α6)	3.03 ± 4.10
CD51 (Integrin αV)	16.62 ± 15.81
CD54 (ICAM-1)	24.79 ± 25.46
CD56 (NCAM)	17.21 ± 18.76
CD69	0.00 ± 0.00
CD73	96.43 ± 3.88
CD90 (Thy-1)	89.87 ± 8.80
CD104 (Integrin β4)	0.00 ± 0.00
CD105	76.99 ± 31.05
CD106 (VCAM-1)	2.11 ± 6.12
CD112 (Nectin)	3.90 + 8.85
CD117 (c-kit)	21.29 ± 34.25
CD133	0.00 ± 0.00
CD140b (PDGFRβ)	76.63 ± 25.00
CD144 (VE-Cadherin)	94.56 ± 3.80
CD146 (MCAM)	24.65 ± 24.97
CD147 (Neurothelin)	69.49 ± 47.52
CD166 (ALCAM)	93.44 ± 10.21
CD184 (CXCR4)	0.00 ± 0.00
CD325 (N-Cadherin)	27.72 ± 37.51
CD338 (ABCG2)	3.45 ± 5.03
HLA-DR	0.00 ± 0.00
VEGFR2	0.00 ± 0.00

Surface markers’ profiling of the *in vitro* cultured CSCMSCs is being shown. High expression of well-known MSC markers such as CD73, CD90, CD105 and CD140b/PDGFRβ was detected. Importantly, CSMSCs showed no measurable expression of hematopoietic and endothelial markers (Data shown are mean ± SD, N = 14).
