# Supplementary material for: Role of Human Corneal Stroma-Derived Mesenchymal-Like Stem Cells in Corneal Immunity and Wound Healing
Source: Sci Rep. 2016 May 19;6:26227. doi: 10.1038/srep26227 (PMC4872602; doi:10.1038/srep26227)
Supplement: Supplementary Figure S1 [file srep26227-s1.pdf]

Role of Human Corneal Stroma-Derived Mesenchymal-Like Stem Cells in Corneal Immunity and Wound Healing

Zoltán Veréb, Szilárd Póliska, RékaAlbert, Ole Kristoffer Olstad, Anita Boratkó, Csilla Csortos, Morten C Moe, Andrea Facskó, Goran Petrovski.

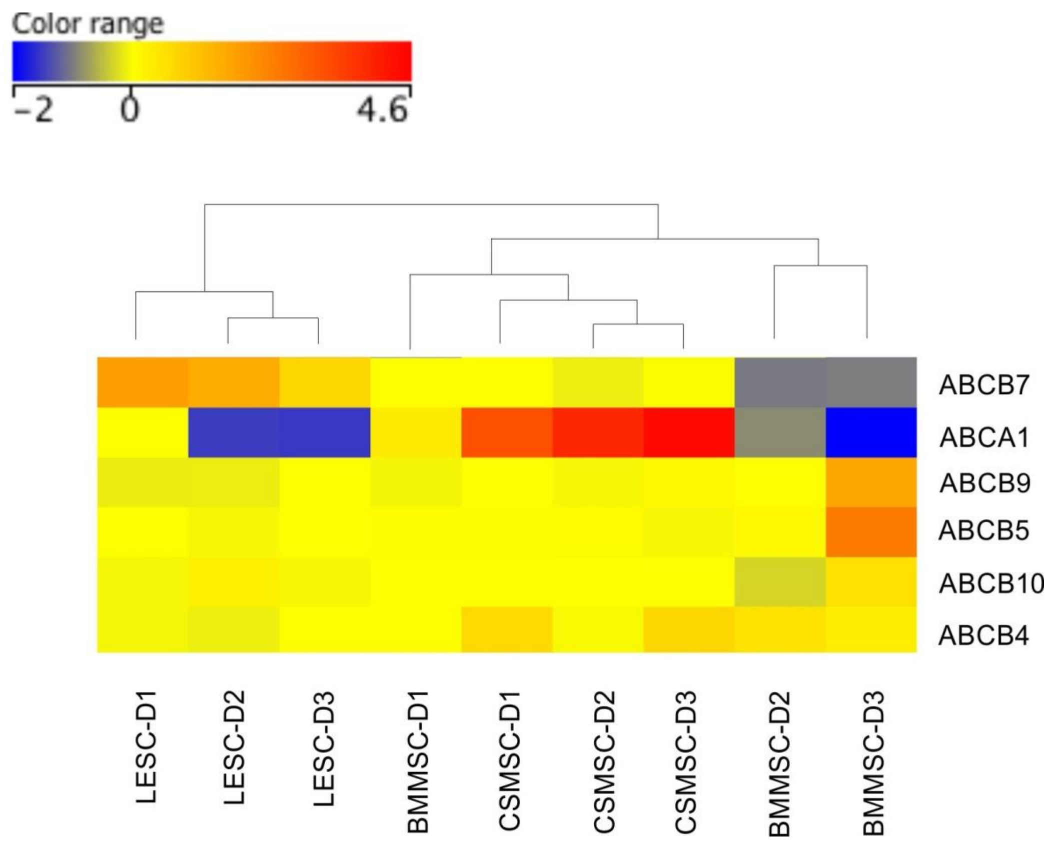

Supplementary Figure S1. Heatmap of the selected ABC transporter B Family gene expression in *in vitro* cultured LESCs, CSMSCs and BMMSCs.

Different expression levels of the transcripts and functional clustering of the ABC transporter Family B genes were expressed in *in vitro* cultured CSMSCs, LESCs and BMMSCs. ABCB5, a previously described putative limbal stem cell marker showed low expression only in one BMMSC donor. Red and yellow colors indicate high and low expression.
